# *Plasmodium vivax* Sub-Patent Infections after Radical Treatment Are Common in Peruvian Patients: Results of a 1-Year Prospective Cohort Study

**DOI:** 10.1371/journal.pone.0016257

**Published:** 2011-01-28

**Authors:** Peter Van den Eede, Veronica E. Soto-Calle, Christopher Delgado, Dionicia Gamboa, Tanilu Grande, Hugo Rodriguez, Alejandro Llanos-Cuentas, Jozef Anné, Umberto D'Alessandro, Annette Erhart

**Affiliations:** 1 Department of Parasitology, Institute of Tropical Medicine, Antwerp, Belgium; 2 Instituto de Medicina Tropical Alexander Von Humboldt, Universidad Peruana Cayetano Heredia, Lima, Peru; 3 Departamento de Bioquimica, Biologia Molecular y Farmacologia, Facultad de Ciencias y Filosofia, Universidad Peruana Cayetano Heredia, Lima, Peru; 4 Multi-Country Malaria Project "Malaria control on the cross border areas of the Andean Region: A community based approach"- PAMAFRO, Organismo Andinode Salud - Convenio Hipolito Unanue, Lima, Peru; 5 Department Microbiology and Immunology, Catholic University of Leuven, Leuven, Belgium; Agency for Science, Technology and Research (A*STAR), Singapore

## Abstract

**Background:**

There is an increasing body of literature reporting treatment failure of the currently recommended radical treatment of *Plasmodium vivax* infections. As *P. vivax* is the main malaria species outside the African continent, emerging tolerance to its radical treatment regime could have major consequences in countries like Peru, where 80% of malaria cases are due to *P. vivax*. Here we describe the results of a 1-year longitudinal follow up of 51 confirmed *P. vivax* patients living around Iquitos, Peruvian Amazon, and treated according to the Peruvian national guidelines.

**Methodology:**

Each month a blood sample for microscopy and later genotyping was systematically collected. Recent exposure to infection was estimated by detecting antibodies against the *P. vivax* circumsporozoite protein (CSP) and all PCR confirmed *P. vivax* infections were genotyped with 16 polymorphic microsatellites.

**Results:**

During a 1-year period, 84 recurrent infections, 22 positive also by microscopy, were identified, with a median survival time to first recurrent infection of 203 days. Most of them (71%) were asymptomatic; in 13 patients the infection persisted undetected by microscopy for several consecutive months. The genotype of mostly recurrent infections differed from that at day 0 while fewer differences were seen between the recurrent infections. The average expected heterozygosity was 0.56. There was strong linkage disequilibrium (*I_A_^s^* = 0.29, p<1.10^−4^) that remained also when analyzing only the unique haplotypes, suggesting common inbreeding.

**Conclusion:**

In Peru, the *P. vivax* recurrent infections were common and displayed a high turnover of parasite genotypes compared to day 0. *Plasmodium vivax* patients, even when treated according to the national guidelines, may still represent an important parasite reservoir that can maintain transmission. Any elimination effort should consider such a hidden reservoir.

## Introduction

In the Peruvian Amazon, after the eradication program was abandoned in the 1980's, malaria cases steadily increased to reach a peak between 1995 and 1998, with 121 268 cases recorded in 1997, more than half due to *Plasmodium falciparum* [1], [2]. Additional efforts of the Peruvian National Malaria Control Program, from 1998 onwards, achieved a substantial reduction of the malaria burden, though this was less pronounced for *Plasmodium vivax* [2], [3]. Indeed, in 2009, among the 25 837 malaria cases recorded in the Loreto department, 85% (n = 21 942) where due to *P. vivax* [4]. The ability to develop liver forms (hypnozoites) that may remain dormant for weeks or even years before relapsing may partly explain, despite vigorous control measures primarily aimed at *P. falciparum,* the importance *P. vivax* has acquired in this setting. Compliance to national treatment strategy for *P. vivax* malaria, three-day chloroquine (CQ) (total dosage 25 mg/kg) and seven-day primaquine (PQ) (total dosage of 210 mg/kg), is often relatively low as symptoms usually disappear after three days while the common occurrence of side effects by PQ reduces the motivation of the patients to complete the treatment [5]. In Brazil, the risk of relapses in travelers after full treatment with CQ (1.5 g over three days) and PQ (210 mg over 14 days) varied between 5 to 25%, with the majority occurring within the first six months [6]. Similarly, in endemic areas in Brazil and Colombia, such risk varied between 6% and 18%, respectively [7], [8], [9]. More recently, in Brazil, 36% patients having received full treatment of 1.5 g CQ and 210 mg of PQ experienced recurrent *P. vivax* infections within 600 days, most of them occurring within the first 180 days [10]. It is unclear if these observations are due to an increased tolerance of *P. vivax* to PQ. Nevertheless, from the above studies there are some indications that *P. vivax* strains from South America responds poorly to the recommended PQ dosage, i.e. 15 mg/day for 14 days. Increasing the dosage to 30 mg/day for 14 days (total dosage of 420 mg) has already been suggested [8].

There is currently little information on the *P. vivax* recurrence rates in the Peruvian Amazon region where transmission is low and clustered and asymptomatic infections are common [2]. In 2003, the *P. vivax* incidence in San Juan district was estimated at 0.39 infections/person/malaria seasons but has probably declined since 2007 thanks to the malaria control efforts [2], [4]. In order to characterize the malaria burden in the Peruvian Amazon and understand the dynamics of *P. vivax* infections, we analyzed blood samples collected during 1-year follow-up in a cohort of patients treated for a *P. vivax* clinical episode. All PCR-confirmed *P. vivax* infections, symptomatic and asymptomatic, were genotyped to determine the local dynamics of *P. vivax* clones in the Peruvian Amazon. Concomitantly, to identify recent inoculations by infected mosquitoes, the presence of antibodies against the circumsporozoite protein (CSP) was measured using the CSP Enzyme-Linked ImmunoSorbent Assay (ELISA). Results are reported below.

## Materials and Methods

### Study sites and population

The study sites are situated at about three to seven kilometers north of Iquitos, on the other bank of the Nanay River (Rio Nanay) and include five neighboring villages, i.e. Manacamiri, Lupuna, Fray Martin, San Pedro and Santa Rita, all being accessible only by boat from Iquitos. These villages are located in a densely forested region with many small pools, rivers and swamps, offering ideal breeding sites for *Anopheles darlingi*, a sylvatic and effective malaria vector [1]–[3]. Its biting activities occur near the breeding sites and throughout the night, with an early peak between 6 pm and midnight [1]. The climate is tropical, and malaria transmission is perennial with a peak during the rainy season, from November to May. Two health posts are located in the study area: one in Manacamiri and the other in Lupuna covering the remaining four villages. The population consists of ‘*mestizos*’ (individuals not belonging to a specific ethnic minority) practicing mainly subsistence farming in forest fields situated at easy walking or paddling distance all year around, with occasional hunting and fishing [5].

### Data collection

The cohort started in March 2008 and *P. vivax* infected patients were recruited by active and passive case detection. Eligible individuals with a *P. vivax* mono-infection were asked to participate after signing (the parent/guardian for minors) an individual informed consent. This study was approved by the Ethics Review Board at Universidad Peruana Cayetano Heredia, Lima, Peru (Project PVIVAX-UPCH, SIDISI code: 053256) and by both the Institutional Review Board of the Institute of Tropical Medicine, Antwerp, and the Ethical Committee of the University Hospital, Antwerp, Belgium. Pregnant women, individuals with known Glucose-6-Phosphate Dehydrogenase deficiency or known adverse events to the treatment, chronic disease or bacterial infection, neuropsychiatric disorders or malnutrition were excluded. All patients included were treated according to the national guidelines, i.e. CQ total 25 mg/kg over three days, and seven days PQ, 0.50 mg/kg/day which started simultaneously with CQ [11]. Patients were visited at home daily by the medical staff attached to the project and the treatment was directly supervised. After drug intake, each patient was observed during one hour. If vomiting occurred during the first 30 minutes, a full dose was re-administered and only half dose, if vomiting occurred 30 minutes after drug intake. After completing the treatment patients were weekly visited until day 28. During these visits blood samples were collected. For the next 11 months, patients were visited monthly at home, regardless of any treatment received during the follow up. At each visit, patients were asked about clinical signs and symptoms, and of any adverse events. A blood sample for microscopy (thick and thin film) and a blood spot on filter paper (Whatman grade 3, Whatman, Springfield Mill, USA) for later molecular (PCR diagnosis and microsatellites genotyping) and serological analysis (CSP ELISA) were systematically collected. Malaria infections detected during the follow up were treated with 3 days of CQ 10 mg/kg 1^st^ and 2^nd^ day and 5 mg/kg 3^rd^ day.

### Laboratory methods

All filter paper blood spots collected at day 0 (before treatment) and during the monthly visits were selected. DNA extraction was performed with the saponine-chelex method and then analyzed by species-specific PCR [12], [13]. Positive samples positive were then selected for genotyping using 16 microsatellites as previously described [14]. The PCR product size was analyzed on a 3730 XL ABI sequencer (Applied Biosystems, Foster city, California, USA). Fragment sizes were determined with Genemapper (Applied Biosystems, Foster city, California, USA) using default microsatellite settings, whereby bands smaller than 100 relative fluorescence units (rfu) were defined as background. For one locus, MS16, the rule was adapted because of stutter: for each sample, only the peaks above 25% of the dominant one (highest rfu) were considered as real alleles [14]. Samples for which we obtained no amplification in some loci were re-analyzed to complete the haplotypes.

The presence of antibodies to the circumsporozoite protein (CSP), expressed during the sporozoite stage, was assessed in patients' sera by Enzyme-Linked ImmunoSorbent Assay (ELISA) using three different long synthetic CS peptides (N-, R- and C-polypeptide) [15]. All three peptides are reported to contain B-cell epitopes and antibodies are generated against these regions in natural infection [15]. The R-polypeptide corresponded to the VK210 type of the CSP protein. One half of the micro plates (Costar EIA/RIA Plate, Corning, New York, USA) were coated with 1 µg/ml peptide and incubated overnight at 4°C. The whole plates were blocked with 5% skim milk in PBS pH 7.4 for two hours at room temperature. After washing, 100 µl of serum samples diluted 1/200 times in PBS-Tween−20 0.05% and 2.5% skimmed milk was added in duplicate on each half of the plate and incubated for one hour at room temperature. Human IgG antibodies bound to the coated peptides were detected by adding 100 µl/well of phosphatase-conjugated polyvalent anti-human immunoglobulin's (Sigma Aldrich, St Louis MO, USA) at a dilution of 1∶1000. The enzymatic activity was developed after incubation for 45 minutes at room temperature with 100 µl/well para-nitrophenyl phosphate substrate (Sigma Aldrich Co., St Louis MO, USA) and stopped with 50 µl/well 12% NaOH. Absorbance was measured at 405 nm in a Microplate Reader (Multiskan Ascent, Thermo Electron Corporation, Vantaa, Finland).

The corrected optical density (OD) of each sample was calculated by subtracting the background OD from the corresponding non-coated wells. Duplicates for which the OD values differed by more than 30% were rejected for the analysis and retested. Otherwise, the median was taken as the final result. Sera from healthy individuals living in Belgium without any history of stay in endemic areas were used as negative controls. The cut-off value for sero-positivity was set as the cumulative mean plus three standard deviations of all the negative control values. Samples were considered positive if the OD of any of the three peptides was higher than the cut-off value.

### Data analysis

Data were entered and cleaned in Excel (Microsoft cooperation, USA) then analyzed with Stata.10 (Stata Release 10, StataCorp, College station, USA). A *P. vivax* recurrence was defined as any *P. vivax* blood infection (symptomatic and asymptomatic) identified after radical treatment by PCR (regardless of symptoms) and occurring between day 28 and month 12. A patient was classified as symptomatic if he/she had fever (≥37.5°C) or history of fever at the time of collection or in the previous 48 hours. *Plasmodium vivax* recurrences were defined as “patent” or “sub-patent” depending on whether the infection was diagnosed either by both microscopy and PCR (patent) or only by PCR (sub-patent). As an estimate of transmission potential due to *P. vivax* carriage, the person-infected month (PIM) rate, i.e. months with *P. vivax* infection (either by microscopy and/or PCR) divided by months of follow-up, was computed and expressed in 100 person-months. Kaplan-Meier survival curves were used to estimate the probability of having a *P. vivax* recurrence in the first year after radical cure treatment. The logrank test was used to compare the different curves.

Prevalence and levels of antibodies against the *P. vivax* CSP were used to determine the risk for new *P. vivax* infections. Recent exposure to *P. vivax* sporozoites was defined as seroconversion (from negative to positive) or, in case of a positive test, at least 50% increase of the corrected OD value between two consecutive monthly samples.

Within each patient, the genetic profile of each recurrent episode was first compared to the one at day-0 and later with all previous episodes as described in [14], and classified into following categories: *i) fully related*: all alleles in all loci of the current infection present in at least one of the previous episodes/infections (including day 0); *ii) incomplete*: as above but with alleles missing in one or more loci; *iii) unrelated*: at least one locus completely different from any of the previous episodes/infections, including day 0.

An infection was defined as polyclonal if there was at least one locus with more than one allele. For each malaria infection, the locus with the highest number of alleles was considered as a proxy for the multiplicity of infection (MOI), representing the minimal number of parasite haplotypes in the sample. The MOI and the average number of allele per locus were assessed for day 0 and samples collected during monthly follow up.

The parasite population characteristics were assessed only in samples with monoclonal infections. The number of alleles/locus, the allelic richness, and the genetic diversity of each locus were computed for each population. Genetic diversity was assessed by calculating the expected heterozygosity (*He*) = [n/(n-1)][1−∑p_i_^2^], where n is the total number of alleles, p_i_ is the frequency of the i^th^ allele in the population. The *He* represents the probability of finding a different allele for a given locus in any pair of haploids randomly drawn from the same population, and it was computed with FSTAT version 2.9.3 [16]. Genetic differentiation *(θ)* between day 0 and monthly follow up samples was measured in FSTAT using the method of Cockerham & Weir [16]. To evaluate the likelihood that identical haplotypes found in two or more samples originated from a different ancestry the p_sex_ values were computed using GenClone ver. 2.0 [17]. This program test whether all samples with identical haplotypes belong to the same genetic clone. The allelic frequencies for a locus was estimated by taking a sample pool composed of all the genotypes distinguished by all loci except the one being estimated and this procedure was repeated for each locus in a round robin fashion. These allelic frequencies were used to calculate the unique genotype probability, as given by *P*_GEN_. The *P*_SEX_ was derived using binomial regression of *P*_GEN_ [18].

The presence of overall multilocus linkage disequilibrium (LD =  non random association of alleles occurring at different loci) was assessed with LIAN software version 3.5 [19]. The Standardized Index of Association (*I_A_^s^*) was estimated as a measure of linkage in the population, and the significance was tested using the Monte Carlo method. The presence of linkage disequilibrium was assessed on monoclonal infections and unique haplotypes.

The distribution of the haplotypes in time was analyzed. The eBURST software (version 3) was used to group haplotypes based on their filiations to each other [20]. Different but related haplotypes were grouped. Two arbitrary criteria to assign haplotypes to different groups were applied, either 15 identical loci out of 16 or at least 11 identical loci [10].

## Results

Fifty-one *P. vivax* patients were recruited between March and May 2008, and followed up monthly for one year. Males (n = 26) and females (n = 25) were equally represented and the median age was 15 years (range: 2–80 years old). The geometric mean of the parasite density at D0 was 1171 parasites/ µl (range 12–9145). During the first 28-day follow-up, no parasite recurrence were observed, except in a 67 years old woman who had at day 28 a sub-patent and asymptomatic *P. vivax* infection that spontaneously cleared before the following monthly visit. All patients but one who left the study area after day 120 completed the 12-month follow up, totaling 604 person-months at risk. Among the 657 follow up samples analyzed, 84 recurrent *P. vivax* infections were identified by PCR, among which only 26% were detected by microscopy (22 patent infections), and even less (18 episodes) were associated with malaria symptoms (fever +/− splenomegaly). Five patients had more than one patent recurrence. The risk of *P. vivax* recurrences (sub- or patent infections) was not correlated with age or sex.

**Figure 1:**
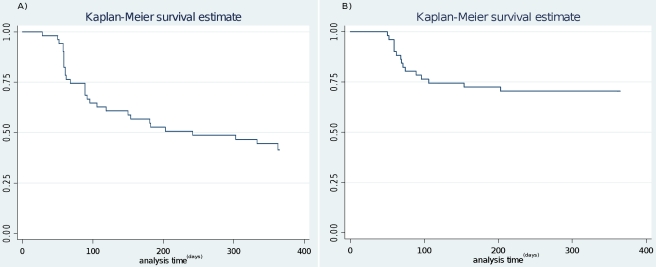
Kaplan-Meier survival analysis showing the probability of remaining free of *P. vivax* infection (A, patent and sub-patent; B, only patent) during one-year of follow up.

**Figure 2:**
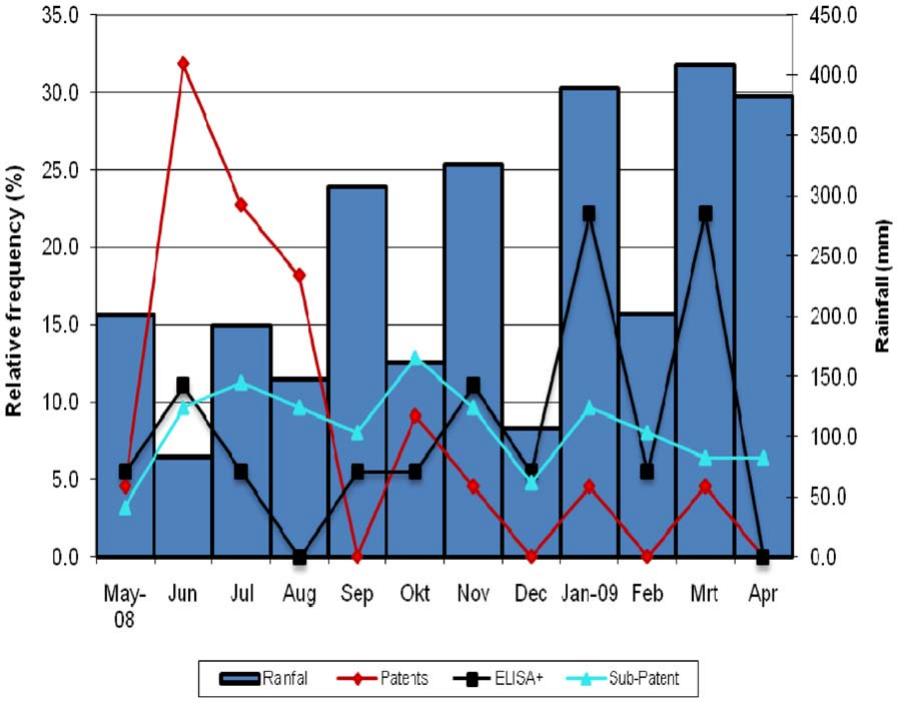
Monthly prevalence of *P. vivax* patent, sub-patent infections and of anti-CSP antibodies, and monthly rainfall.

No recurrent infections were observed in 22 (43%, 22/51) patients (median age 16 years old). Among the 29 patients (57%, 29/51, median age 15 years old) who experienced a total of 84 *P. vivax* recurrences, 13 had one episode and 16 more than one, with 3 patients with up to seven recurrences. About half of the patients with recurrent infections (14/29) had them over two or more consecutive months, all of them sub-patent and asymptomatic (Table 1). In three patients the sub-patent infection was followed by a patent infection within the following month while in all other patients the infection was cleared without treatment (Table 1). The overall person infected month (PIM) rate was estimated at 13 per 100 person-months, i.e. an average 1.6 months of infection. When considering only the 29 patients with recurrences, the PIM was 22.7/100 person-months, i.e. an average of 2.7 months of infection. The risk (Kaplan Meier estimate, KME) of having a *P. vivax* recurrence (patent and sub-patent) after one year was 59%, with 203 days as the median survival time to first recurrence. When considering only patent infections the risk was 30% (Figure 1) with half of the primary patent recurrences occurring within the first 70 days of follow up. Patent recurrent infections tended to occur mainly during month two to four, with a tendency to decrease with time, while sub-patent infections were observed throughout the whole follow up period (Figure 2). The risk of recurrence was higher in Santa Rita (KME risk  = 69%, median time  = 154 days) than in the other four villages (KME risk  = 53%, median time: 362 days) (logrank test p = 0.34, α = 0.05). Though not significant, this difference may be due to the non-availability of Insecticide-treated nets in Santa Rita at the time the study was carried out.

**Table 1:** Chronogram of the 29 patients with *P. vivax* recurrences.

		2008										2009			
Patient	Village	Mar	Apr	May	Jun	Jul	Aug	Sep	Oct	Nov	Dec	Jan	Feb	Mar	Apr
1	Manacamiri	**X**								**S**					
2	Manacamiri		**X**												**S**
3	Manacamiri	**X**				**S**		**S**		**S**		**S**			
4	Manacamiri	**X**			**S**										
5	Manacamiri			**X**		**P**			**S**	**S**	**S**	**S** *	**S**		
6	Manacamiri		**X**		**P**		**S**	**S**					**S**		**S**
7	Manacamiri	**X**		**S**	**S**	**S**	**S**	**S**	**S**			**S** *			
8	Manacamiri		**X**		**S**										
9	Manacamiri		**X**						**S**						
10	Manacamiri		**X**			**P** *									
11	Lupuna		**X**		**P**		**P**	**S**							
12	Lupuna		**X**			**S**	**S**								
13	Lupuna		**X**		**P**										
14	Fray Martin		**X**			**P**	**S**								
15	Fray Martin		**X**		**P** *				**S** *	**S**				**S** *	**S**
16	Fray Martin		**X**		**P**										
17	San Pedro		**X**						**S**						
18	San Pedro		**X**	**P** *		**P**			**P**						
19	Santa Rita	**X**		**S**		**S**	**S**			**S** *					
20	Santa Rita	**X**			**P**		**S**	**S**	**P** *						
21	Santa Rita	**X**					**P**		**S**	**S**	**S** *	**S**	**S**	**S**	
22	Santa Rita	**X**										**S** *	**S**	**S** *	
23	Santa Rita		**X**		**S**	**P**								**P** *	
24	Santa Rita			**X** *										**S** *	
25	Santa Rita			**X**			**P**								
26	Santa Rita		**X**							**P** *					
27	Santa Rita		**X**					**S**	**S**		**S**	**S**	**S** *		**S**
28	Santa Rita			**X**	**P** *	**S**	**P**					**P** *	**S**		
29	Santa Rita			**X**					**S**						

X =  time of enrolment in the study, S  =  sub-patent infection, P  =  patent infections,

* =  indicate positive ELISA indicating a recent sporozoite inoculation. Two patients had a positive CSP ELISA at day 0 (April) though no recurrent infections were reported.

### Complexity of the parasite population

The median expected heterozygosity (*He*) was 0.56, with a wide range of diversity in the different loci (ranging from 0 to 0.84) (Table 2). The population genotyping characteristics of day 0 samples have already been published elsewhere [21]. The *He* for samples obtained during the follow up were comparable to those obtained for day 0 though the monthly follow up samples carried additional alleles at MS8 and MS16 as also indicated by the allelic richness (Table 2). This probably explains the small but significant genetic difference (*θ* = 0.015, p = 0.0027) between day 0 and monthly follow up samples.

**Table 2:** Genetic diversity of the 135 positive *P. vivax* samples analyzed.

	Gene diversity *(He*°*)*			Allelic Richness		
Locus	Full	D0*	MF	Full	D0	MF
MS1	0.10	0,20	0.04	2.99	2,00	1.98
MS2	0.68	0,65	0.69	4.00	3,99	4.00
MS3	0.61	0,67	0.56	3.98	3,00	3.95
MS4	0.44	0,36	0.49	4.00	3,00	4.00
MS5	0.28	0,27	0.29	2.00	2,99	2.00
MS6	0.62	0,64	0.62	5.96	5,00	4.94
MS7	0.00	0,00	0.00	1.00	1,00	1.00
MS8	0.84	0,74	0.84	12.00	8,00	9.00
MS9	0.77	0,74	0.76	7.00	6,00	6.00
MS10	0.59	0,53	0.63	5.00	4,00	4.00
MS12	0.68	0,64	0.70	6.93	6,00	4.00
MS15	0.51	0,51	0.51	5.98	5,98	4.00
MS16	0.65	0,71	0.62	11.90	5,99	8.82
MS20	0.72	0,75	0.71	6.95	5,00	5.86
Pv6635	0.75	0,72	0.76	8.00	6,00	7.00
Pvsal	0.67	0,69	0.65	7.99	6,00	7.90
**Average**	**0,56**	**0,55**	**0.55**	**5.98**	**4,62**	**4.90**

*Already published in [21]. MF = monthly follow-up. °*He*: expected heterozygosity.

**Figure 3:**
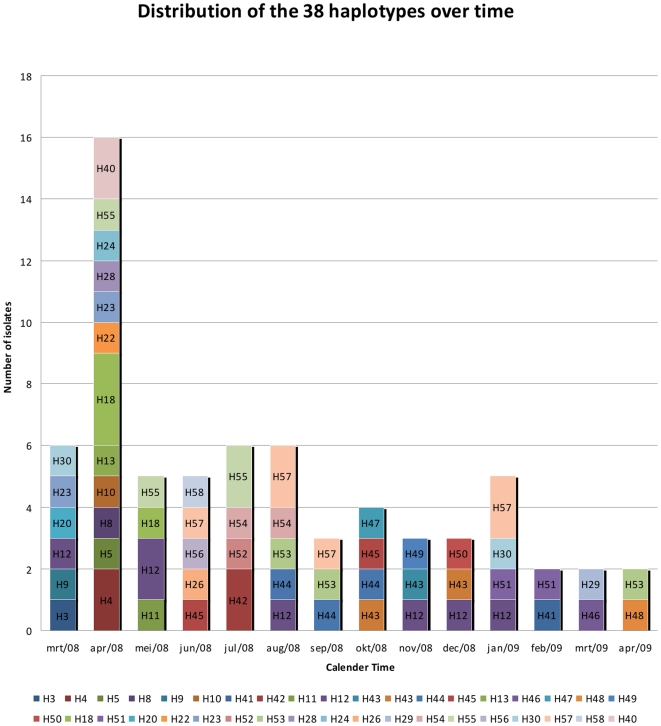
Distribution of the 38 distinct haplotypes over the different episodes (n = 68) by calendar month.

Only seven polyclonal infections at day 0 and three during the follow up period were observed. Most infections were monoclonal, suggesting little outbreeding. Indeed, strong linkage disequilibrium (*I^S^_A_* = 0.29, p<1.10^−4^) was observed and remained significant when considering only the unique haplotypes (*I^S^_A_* = 0.21, p<1.10^−4^). From the monoclonal samples, 68 had complete genotypes among which 38 distinct haplotypes were identified. The frequencies of the individual haplotypes was low with H12 having the highest frequency (10%). Twenty-three haplotypes occurred only once (Figure 3) while 15 haplotypes were observed in more than one malaria episode. From these 15 haplotypes 12 were spread over different months, suggesting little temporal clustering. The probability of having identical haplotypes resulting from a different sexual reproduction was low (average p_sex_ = 5.25×10^−4^), indicating the existence of a common ancestor and a clonal propagation as result of inbreeding. The genetic relatedness among haplotypes was calculated with eBURST. Five clusters of related haplotypes and 20 singletons were identified when the haplotypes within these clusters carry identical alleles in at least 15 loci and decreased to three clusters and six singletons when the criterion of at least 11 loci was applied [10]. No temporal clustering for related haplotypes was observed.

For the remaining infections (n = 67), the haplotypes could not be retrieved as they were either polyclonal (n = 10) or no allele was obtained (n = 57) for one or more loci, even after repeating the PCR. No amplification could be obtained for 13 of the 84 recurrent infections, all of them sub-patent and asymptomatic, despite a positive species specific PCR.

### Equality *versus* heterology of recurrent infections

Out of the 71 recurrent infections for which genotyping data was obtained, 82% (n = 58) carried alleles different from those at day 0. Only three recurrent infections had a fully related genotype with the infection at day 0. All fully related infections occurred within the first 6 months of follow up. In 10 infections, the genotypes were incomplete, though the alleles present were identical to those at day 0. The incomplete genotypes were analyzed assuming that all incomplete genotypes were either a) fully related or b) different compared to the genotype obtained at day 0. Consequently, the proportion of recurrences with a different genotype ranged between 82% and 96%. Out of the 22 patent infections, 18 were different from those at day 0.

In 30 recurrent episodes (42%, 30/71), the genotype differed within the same patient from all previous infections; 18 occurred during the first recurrent episode and within the first three months of follow up. Seventeen episodes (24%, 17/71) had a similar genotype with one of the earlier *P. vivax* episode, while for 24 infections (34%) incomplete haplotypes were obtained in which all present alleles were similar to all previous infections. When assuming that incomplete haplotypes were either fully related or different, the proportion of recurrences with genotypes different from all previous infections ranged between 42% and 76%. When considering only patent infections (n = 22), 16 (73%) were different from all previous infections while six were fully related to one or more previous infections.

### Antibody prevalence against the circumsporozoite protein

All blood samples PCR positive for *P. vivax* were analyzed to detect the presence of anti-CSP antibodies. The OD values were generally low (0.07–1.3); 6% (3/51) samples collected at day 0 and 21.4% (18/84) at the time of recurrent infection were positive for anti-CSP antibodies. No significant association between anti-CSP antibodies and unrelated genotypes, sex or age was found. The majority of positive samples (60%, 11/18 during follow up and 2/3 at day 0) had been collected in individuals from Santa Rita. Seroprevalence increased with increasing rainfall while patent infections showed an opposite trend, suggesting that during the early months of follow up the observed recurrences may have been relapses (Figure 2).

## Discussion

Despite the low transmission, a substantial number of recurrent *P. vivax* infections (patent and sub-patent) were observed after the supervised administration of the recommended radical cure regimen. Unlike previous *P. vivax* cohort studies in which blood samples were collected only on symptomatic individuals [10], [14], [22]–[25], our cohort patients were systematically examined every month and blood samples collected regardless of symptoms, allowing for the detection of asymptomatic infections, a common occurrence in the Amazon Basin [2], [3], [26]–[28]. Microscopy detected only 26% of the infections identified by PCR. In regions were sub-patent recurrences are frequent, evaluation of treatment efficacy based on clinical signs and microscopy might be insufficient and likely underestimate the true number of recurrent infections. Consequently the human reservoir is probably much larger than previously thought. As indicated by the high proportion of sub-patent and asymptomatic infections, partial immunity could be induced despite the low transmission. Moreover, in several patients *P. vivax* infections remained sub-patent and asymptomatic for several consecutive months until spontaneous clearance without treatment. In only three patients the sub-patent infection became patent and was treated. The median survival time to the first relapse was about six months when considering all infections and the majority of patent infections occurred within three months of follow up, which is consistent with relapse patterns previously reported in Latin America [6], [9], [10], [29]. The substantial proportion of individuals (43%, 22/51) in the cohort who never experienced a recurrent infection throughout the follow up period suggests either a heterogeneous exposure or risk to *P. vivax* infections or an infection with PQ susceptible parasites with corresponding clearance of hypnozoites [2], [30], [31]. Alternatively, recurrent infections in these individuals may have been missed by the monthly sampling and cleared by the immune system before being detected.

Although, comparison between different studies is difficult, given the variation in methodology, the observed parasite population diversity and polyclonality was lower than previously reported in 2003 and 2006 for parasite populations in the same region of Peru and in the bordering Brazilian Amazon province [10], [21], [22], [31]. For the latter, the difference could be explained by the variability of malaria endemicity found in the Amazon region [27], [28], [30]. In Peru, we observed a lower diversity and polyclonality of the parasite population in 2008 compared to previous studies done in 2003 and 2006. This could be explained by the decreasing trend in malaria incidence [4], [21], [31]. However, the difference with the 2003 study may also be due to the different molecular markers used for the genotyping [31].

The strong linkage disequilibrium observed suggests little out-breeding as a result of the low transmission and the paucity of polyclonal infections [21]. Structuring our haplotypes with eBURST showed relatively few groups and singletons, indicating that the circulating haplotypes are closely related to each other and support the clonal population structure. The absence of temporal clustering of specific haplotypes could be explained by the little outbreeding, the activation of hypnozoites and by the presence for long periods of undetected sub-patent infections. This contrasts with the situation described in the Brazilian Amazon where temporal clustering occurs [10], [22].

Despite, the clonal population structure, a small though significant genetic differentiation between monthly follow up and day 0 was found, possibly because recurrent infections carried new alleles not present or detected at day 0, in MS8 and MS16 as indicated also by the allelic richness. Heterologous activation of hypnozoites or new infections may explain this observation. The majority of recurrent infections carried a genotype different from that at day 0 while fewer differences were observed when comparing with all previous episodes within the individual patient.

Several individuals carried sub-patent *P. vivax* infections for several months, many of them with the same haplotype, possibly due to a single parasite clone. However, in three of these individuals new haplotypes occurred, without any indication of exposure to new infection, i.e. no anti-CSP antibodies were found, suggesting that these infections may have originated from liver forms. However, the sensitivity of the CSP ELISA test is not optimal (60–77%) and exposure to new infections cannot be categorically ruled out [32]–[34]. Indeed, the OD of the CSP positive samples were generally weak, an expected result when considering the low number of sporozoites per inoculation and the short time they circulate in the blood stream [33]. In addition, the peptide which corresponded to the central repeat domain is analogue to the VK210 type of CSP, while in the Amazonian regions the two other variant, VK247 and *P. vivax*-like, are present [35], [36]. Nevertheless, positivity corresponded to the malaria transmission dynamics, indicating that it could be used as a proxy for transmission intensity [37]–[40]. The use of CSP ELISA as an indicator of individual exposure to infection may be more questionable as there was no correlation between CSP positivity and occurrence of different haplotypes. This could be due to activation of heterologous hypnozoites while new infections could carry similar genotypes given the observed clonal population structure.

Currently, no data is available on the length of time sub-patent infection remain circulating in the blood stream and on the role of new inoculations and hypnozoites to maintain them. Probably, the balance between the natural acquired immunity and parasite factors such as its antigenic variation is important. The clonal population structure with the low parasite population diversity probably contributed to the suggested rapid acquisition of immunity. The exact role of asymptomatic/sub-patent carriage in maintaining the transmission needs to be further evaluated. Though the capability of asymptomatic carriers to transmit is 22-fold lower than in symptomatic individuals, the chronic nature and the larger reservoir of the asymptomatic infections could compensate the lower transmissibility [41].

The proportion of incomplete haplotypes in this study was substantial and can be explained by the numerous sub-patent infections whose density was so low that they could not be fully genotyped. Therefore the establishing with certainty their relation with previous infections was not possible. This was an important limiting factor for the analysis of genotype relatedness within a patient's subsequent episodes. Whole genome amplification before genotyping or a nested PCR protocol could improve the sensitivity of the test and possibly reduce the proportion of incomplete haplotypes [42], [43].

Treatment efficacy at day 28 was high; just one patient had a sub-patent infection that was identified only retrospectively and hence not treated. Nevertheless, the following month this patient did not have any infection as detected by PCR, indicating that the day 28 infection had been successfully treated. However, the absence of infection at day 28 does not necessarily exclude CQ resistance, low levels of tolerant parasites might still be cleared by the host immunity. Chloroquine resistant *P. vivax* cases have been observed in Peru [44] and the recurrent infections beyond day 28 with the genotype fully related to day 0 may suggest that some parasites may be CQ resistant. However, it is impossible to make this statement with certainty because CQ plasma levels for our cohort are not available. The observed clonal population structure in Peru might represent a risk for the rise and spread of drug resistance in this area [45].

In conclusion, despite supervised radical cure treatment, several patients experienced over a 1-year period *P. vivax* recurrent infections, most of them asymptomatic and even sub-patent. Multilocus genotyping was difficult because of the low parasite densities. Nevertheless, infections with the same or related haplotypes were not clustered in time and were shared among different episodes and patients, a finding probably due to their chronic nature. Moreover, analysis of the parasite population structure suggested little out-breeding with few polyclonal infections, indicating low transmission. Indeed, ELISA results correlated well with the low risk of infection at population but not at individual level. Further optimization and validation of the ELISA test is needed. Serology and PCR based tools could be helpful in areas like Peru where, despite appropriate treatment, individuals with a *P. vivax* infection may still represent an important parasite reservoir for maintaining transmission. Any elimination effort should consider such a hidden reservoir.
